# Lung Fibroblasts Take up Breast Cancer Cell-derived Extracellular Vesicles Partially Through MEK2-dependent Macropinocytosis

**DOI:** 10.1158/2767-9764.CRC-23-0316

**Published:** 2024-01-22

**Authors:** Yuhao Wan, Yue Zhao, Minghui Cao, Jingyi Wang, Sheila V. Tran, Zhixuan Song, Brent W. Hsueh, Shizhen Emily Wang

**Affiliations:** 1 Department of Pathology, University of California San Diego, La Jolla, California.

## Abstract

Extracellular vesicles (EV) have emerged as critical effectors in the cross-talk between cancer and normal cells by transferring intracellular materials between adjacent or distant cells. Previous studies have begun to elucidate how cancer cells, by secreting EVs, adapt normal cells at a metastatic site to facilitate cancer cell metastasis. In this study, we utilized a high-content microscopic screening platform to investigate the mechanisms of EV uptake by primary lung fibroblasts. A selected library containing 90 FDA-approved anticancer drugs was screened for the effect on fibroblast uptake of EVs from MDA-MB-231 breast cancer cells. Among the drugs identified to inhibit EV uptake without exerting significant cytotoxicity, we validated the dose-dependent effect of Trametinib (a MEK1/2 inhibitor) and Copanlisib (a PI3K inhibitor). Trametinib suppressed macropinocytosis in lung fibroblasts and inhibited EV uptake with a higher potency comparing with Copanlisib. Gene knockdown and overexpression studies demonstrated that uptake of MDA-MB-231 EVs by lung fibroblasts required MEK2. These findings provide important insights into the mechanisms underlying lung fibroblast uptake of breast cancer cell-derived EVs, which could play a role in breast cancer metastasis to the lungs and suggest potential therapeutic targets for preventing or treating this deadly disease.

**Significance:**

Through a phenotypic screen, we found that MEK inhibitor Trametinib suppressed EV uptake and macropinocytosis in lung fibroblasts, and that EV uptake is mediated by MEK2 in these cells. Our results suggest that MEK2 inhibition could serve as a strategy to block cancer EV uptake by lung fibroblasts.

## Introduction

Extracellular vesicles (EV), including exosomes of endocytic origin and microvesicles shed from plasma membranes, mediate the cross-talk between cancer and normal cells through local and long-range transfer of functional cargo including signaling nucleic acids and proteins. Cancer cell–secreted EVs contribute to tumor heterogeneity and plasticity, resistance to therapies, vascular remodeling, immunomodulation, and establishment of premetastatic niches (1, 2). Circulating EV-based biomarkers are being exploited for risk prediction, early diagnosis, and prognosis of human diseases including cancer (3–5). Increased cancer EV cargo, such as miRNAs, in the circulation can be detected at a premetastatic stage and correlate with metastasis in patients with breast cancer. Systemic inhibition of certain EV cargo derived from cancer cells suppresses metastasis and other systemic effects in preclinical studies (4–7). Therefore, targeting cancer cell-derived EVs could be a new angle in cancer therapy.

Currently, there are no approved EV-targeted drugs as an adjuvant therapy for treatment of cancer or other diseases. Experimental strategies targeting the pathogenic functions of tumor-derived EVs include suppressing EV secretion by cancer cells (at the origin), blocking EV uptake by normal cells (at the destination), depleting EVs from the circulation via hemodialysis (“*en route*”), and inhibiting the signaling cascade of specific EV cargo (5). Because of the versatile functions of EVs mediated by a broad variety of cargo molecules, it would be more effective to block the trafficking of EVs than to target individual EV cargo effectors. Dynasore, an endocytosis inhibitor targeting dynamin, suppresses the endocytic biogenesis of a certain subtype of EVs (exosomes) and EV uptake in some cells (8, 9). However, dynamin is required for the endocytosis of many critical signaling molecules such as receptor tyrosine kinases, G-protein–coupled receptors, and synaptic vesicle components (10). In addition, dynasore is often used at a high *in vitro* concentration (such as 80 µmol/L) to exert an effect on EVs, and it also exerts dynamin-independent effects such as reducing cholesterol in the plasma membrane and disrupting lipid raft organization (11). These drawbacks limit the therapeutic use of dynasore.

In this study, we carried out a phenotypic screen to identify compounds that suppressed the uptake of breast cancer cell-derived EVs by lung fibroblasts. We focused on lung fibroblasts because EVs from breast cancer cells have been shown to be taken up by lung fibroblasts *in vivo* and promote lung metastasis through a variety of mechanisms (7, 12). The compounds were selected from the NCI Development Therapeutics Program's Approved Oncology Drugs Set consisting of 179 FDA-approved anticancer drugs to focus on targeted therapies especially kinase inhibitors. Our results suggest MEK2 inhibition as a potential strategy to block cancer EV uptake by lung fibroblasts.

## Materials and Methods

### Chemical Reagents

Compounds used for screening were selected from the Approved Oncology Drugs Set obtained from the NCI Development Therapeutics Program and listed in Supplementary Table S1. 5-(N-ethyl-N-isopropyl)amirolide (EIPA; catalog no. 337810), chlorpromazine (CPZ; catalog no. J63659-09), Genistein (catalog no. 328271000), Transferrin-Fluorescein (catalog no. T2871), BSA-Alexa 488 (catalog no. A13100), and Dextran-Fluorescein (catalog no. D1823) were purchased from Thermo Fisher Scientific. The human MEK1 (MAP2K1) siRNA #1 and #2 (Assay ID # 324 and 325) and human MEK2 (MAP2K2) siRNA #1 and #2 (Assay ID # 1080 and 1081) were purchased from Invitrogen. The control siRNA was purchased from Cell Signaling Technology (catalog no. 6201S). Plasmids encoding human wild-type MEK2 or a catalytically inactive mutant (K101A), LZRS-Mek2-wt (Addgene plasmid # 21201; https://n2t.net/addgene:21201; RRID:Addgene_21201) and LZRS-Mek2-K101A (Addgene plasmid # 21191; https://n2t.net/addgene:21191; RRID:Addgene_21191), were gifts from Paul Khavari.

### Cell Culture

Human breast cancer cell lines MDA-MB-231 (HTB-26) and MDA-MB-468 (HTB-132), murine mammary tumor cell line 4T1 (CRL-2539), murine fibroblast cell line NIH3T3 (CRL-1658), and primary human lung fibroblasts were obtained from the ATCC. MDA-MB-231, MDA-MB-468, 4T1, and NIH3T3 cells were cultured in DMEM (Gibco) supplemented with 10% FBS (Sigma-Aldrich). Primary human lung fibroblasts were obtained from ATCC (catalog no. PCS-201-013) and cultured in Fibroblast Basal Medium (ATCC; catalog no. PCS-201-030) supplemented with Fibroblast Growth Kit-Low serum (ATCC; catalog no. PCS-201-041) or Fibroblast Growth Kit-Serum-free (ATCC; catalog no. PCS-201-040). Overexpression of a membrane-targeted Lck-GFP for EV labeling is described in our previous study (13). Cell lines were authenticated by ATCC short tandem repeat profiling and routine examination of morphology and consistent *in vitro* growth properties. Cell lines were grown for no more than 15 passages after thawing. Cell lines were periodically tested and confirmed negative for *Mycoplasma* contamination (last tested: January 2023) using a PCR-based method described in ref. 14.

### EV Purification

MDA-MB-231 EVs were purified from conditioned medium (CM) using differential centrifugation. Briefly, CM was collected from cultured cells grown in serum-free DMEM containing 1 × antibiotic-antimycotic (Gibco) for 24 hours and precleared by centrifugation at 500 × *g* for 15 minutes and then at 10,000 × *g* for 20 minutes. EVs were then passed through a 0.22-µm filter and pelleted by ultracentrifugation at 110,000 × *g* for 120 minutes and washed in PBS using the same ultracentrifugation conditions.

### EV Characterization

To quantify EVs based on protein amount, bicinchoninic acid (BCA) assay was used. Briefly, RIPA Lysis and Extraction Buffer (Thermo Fisher Scientific, catalog no. 89901) was added to EV-containing solution at 1:1 ratio and incubated on ice for 15 minutes. The mixture was centrifuged at 14,000 × *g* for 15 minutes, and 10 µL of the supernatant was used as input for BCA assay following manufacturer's protocol (Pierce BCA Protein Assay Kit; Thermo Fisher Scientific, catalog no. 23225). Nanoparticle Tracking Analysis was performed to determine the size distribution and concentration of nanoparticles by using a NanoSight NS300 (Malvern Panalytical).

### Fluorescent Staining of EVs

After the first ultracentrifugation, EV pellet resuspended in PBS was passed through a 29 G needle for at least five times in order to break down large aggregates. Then, DiI (1,1′-Dioctadecyl-3,3,3′,3′- Tetramethylindocarbocyanine Perchlorate; Invitrogen), or DiO when indicated, was added to EV solution at 5 µmol/L and incubated for 30 minutes at 37°C. Meantime, another vial containing PBS and 5 µmol/L DiI but no EVs was incubated in parallel and later served as the “Dyeonly control”. Both the EV and control samples were then centrifuged at 2,000 × *g* for 10 minutes to remove large DiI micelles. Both samples were then concentrated using a 100 kD MWCO ultrafiltration unit (Thermo Fisher Scientific; catalog no. 88533), followed by another centrifugation at 10,000 × *g* for 10 minutes to remove smaller DiI micelles. The fluorescence intensity of stained EVs was measured using a Varioskan LUX multimode microplate reader (Thermo Fisher Scientific) and stored at −80°C till use.

### EV Uptake Microscopy

Cellvis 96-well glass-bottom plates (Cellvis, catalog no. P96-1.5H-N) were precoated with collagen I (Gibco, catalog no. A1048301) according to the manufacturer's protocol. Normal primary human lung fibroblasts within 10 passages were prestained with CFSE (5-(and 6)-Carboxyfluorescein diacetate succinimidyl ester of CFDA SE; BioLegend; catalog no. 423801) following the manufacturer's protocol. The prestained cells were seeded at a density of 5,000 cells per well in serum-containing medium. After the cells attached, they were washed once with PBS and changed to serum-free medium for an incubation of 24 hours in the presence or absence of compounds as indicated. For compound library screen, the compounds were tested at the final concentration of 200 nmol/L. Next, serum-free medium containing approximately 1 µg of DiI-stained EVs was added to each well, and the plate was incubated at 37°C with 5% CO_2_ for 6 hours to allow EV uptake by the lung fibroblasts. Following the incubation, the cells were washed three times with PBS, and then fixed in a 4% paraformaldehyde (PFA) solution (Thermo Fisher Scientific, catalog no. J19943.K2) at room temperature for 15 minutes. The cells were then washed three times with PBS. To visualize the cell nuclei, the cells were stained with 2 µmol/L Hoechst 33342 (Thermo Fisher Scientific; catalog no. 62249) for 5 minutes at room temperature. Excess dye was removed by washing the cells three times with PBS. Finally, stained cells were stored in PBS at 4°C before high-content microscopy. Cells were imaged using a Nikon Eclipse Ti2-E BioPipeline system equipped with a Lumencor Celesta laser engine and a spinning disk scanner (X-Light V3, CrestOptics) and a Plan Apo lambda 10 × NA 0.45 air objective. The lasers used were 405, 477, and 546 nm, and the emission wavelength and bandwidth of the three channels used were 450/40, 520/40, and 590/50 nm. The images are acquired with an ORCA-Fusion digital CMOS camera (Hamamtsu). Illumination, automated image acquisition and processing were controlled by NIS Elements Advanced Research software JOBS module (Nikon Instruments) and Nikon's automatic image processing pipeline (GA3). Briefly, a threshold set based on background fluorescent intensity was added to all channels to remove the noise. Binary images of CFSE signals and Hoechst 33342 signals were used to define cell objects and nuclei objects, respectively, whereas the binary image of DiI signals was used to mask the original image. Watershed algorithm was used to combine cell objects and nuclei objects such that single cells were delineated. Finally, DiI signals (after masking) were quantified in every single-cell object previously defined. Results were exported in excel format.

### Flow Cytometry

Primary lung fibroblasts cultured and treated on 12-well plates were detached through trypsinization, fixed with 4% PFA in PBS, and analyzed using a BD FACSCanto RUO cytometer (BD Biosciences) and FlowJo software (10.8.1).

### RNA Extraction, Reverse Transcription, and qPCR

These procedures were carried out as reported previously (7, 9). Sequences of the primers were obtained from PrimerBank (https://pga.mgh.harvard.edu/primerbank/). For human FN1, a forward primer 5′-CGGTGGCTGTCAGTCAAAG and a reverse primer 5′-AAACCTCGGCTTCCTCCATAA were used. For human S100A4, a forward primer 5′-GATGAGCAACTTGGACAGCAA and a reverse primer 5′-CTGGGCTGCTTATCTGGGAAG were used. GAPDH was detected using primers 5′-GGAGCGAGATCCCTCCAAAAT and 5′-GGCTGTTGTCATACTTCTCATGG and served as an internal reference to calculate the relative level of each mRNA. Reverse transcription was performed using random primers. PCR data were collected and analyzed using Bio-Rad CFX Manager software (Bio-Rad, version 3.1).

### Transfection of siRNA or Plasmid DNA

Transfections of primary lung fibroblasts were performed using Lipofectamine 2000 (for DNA) or RNAiMAX (for siRNA) Transfection Reagent (Thermo Fisher Scientific, catalog nos. 11668027 and 13778150). Forty-eight hours later, transfected cells were analyzed by Western blots or treated with EVs for uptake assay.

### Western Blots

Cells were lysed in RIPA Lysis and Extraction Buffer supplemented with cOmplete Protease Inhibitor Cocktail (Roche, catalog no. 04693124001) and Halt Phosphatase Inhibitor Single-Use Cocktail (Thermo Fisher Scientific, catalog no. 78428). Protein extracts were then separated by electrophoresis on a 4%–15% precast SDS polyacrylamide gel (Bio-Rad, catalog no. 4561084 or 4561086). Separated proteins in the gel were then transferred onto a polyvinylidene difluoride membrane (Bio-Rad, catalog no. 1620177) under 100 V for 75 minutes. After transfer, blot was blocked with 5% nonfat dry milk in TBS with 0.1% Tween 20 detergent (TBST) and then stained with the primary antibody. Horseradish peroxidase–conjugated secondary antibodies were used for all Western blots. Signals were detected using Pierce ECL Western Blotting Substrate (Thermo Fisher Scientific, catalog no. 32106). Information of the primary antibodies is included in Supplementary Table S2.

### Statistical Analysis and Reproducibility

Quantitative data are presented as mean ± SD unless noted otherwise. Two-tailed Student *t* tests were used for comparison of means of data between two groups. For multiple independent groups, one-way ANOVA with *post hoc* Tukey tests were used. Values of *P* < 0.05 were considered significant. All samples that have received the proper procedures with confidence were included for the analyses.

### Data Availability

The data generated in this study are available upon request from the corresponding author.

## Results

### Setting up a Microscopic Assay for Quantitative Measurement of EV Uptake

In this study, we started with EVs from the MDA-MB-231 metastatic breast cancer cell line. Previous studies have shown that the MDA-MB-231 EVs can enter and influence the function of a broad range of non-cancer cells including fibroblasts from various tissues (9, 12, 15). EVs prepared from the CM of MDA-MB-231 by differential centrifugation contained enriched protein markers of EVs (16), including transmembrane proteins CD9 and CD63 and cytosolic proteins Alix and TSG101, but lacked a *cis*-Golgi matrix protein GM130 when compared with the whole-cell lysate (Supplementary Fig. S1A). These EVs exhibited a typical size distribution of small EVs, ranging from 40 to 150 nm with a mean diameter of 115.8 nm (Supplementary Fig. S1B).

**FIGURE 1:**
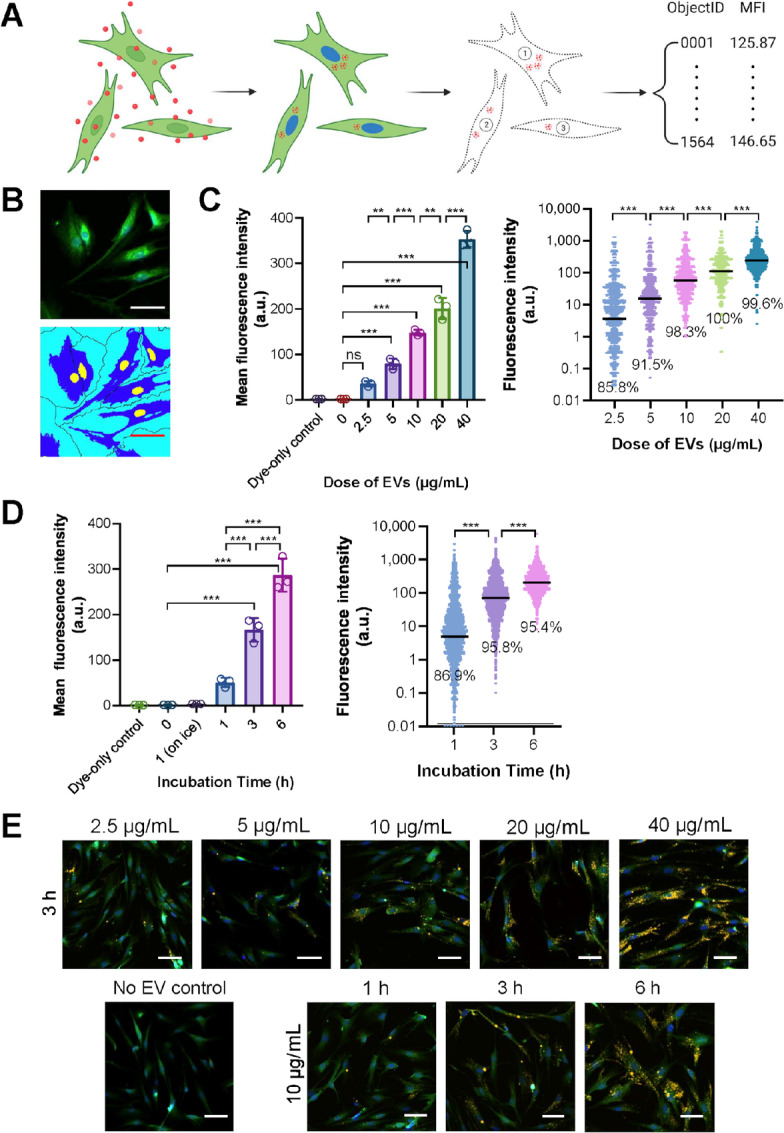
The high-content microscopic platform for quantitative measurement of EV uptake. **A,** A schematic graph showing how cellular uptake of fluorescently-labeled EVs was detected and quantified. Briefly, cells were prestained with CFSE (green) and EVs were prestained with DiI (red). Following the incubation, extracellular EVs were washed off and cell nuclei were stained with Hoechst 33342 (blue). During fluorescent image analysis, the CFSE and Hoechst 33342 signals were used to delineate single-cell boundaries and the DiI signals per cell were thereafter quantified. ObjectID, ID for single cells. MFI, mean fluorescent intensity. **B,** Representative images showing the single-cell delineation process. Human lung fibroblasts were captured at 10 ×. Scale bar, 100 µm. **C,** Dose-dependent uptake of MDA-MB-231 EVs by human lung fibroblasts. Indicated dosages of EVs were added to cells growing in 100 µL of medium on a 96-well plate for an incubation of 3 hours. Five independent images were taken from each well. The results are presented as the calculated mean value of all MFI in a given well (left; *n* = 3 independent wells; data shown as mean ± SD) or as the individual MFI of each single cell in that group (right; *n* > 1,000 cells). In the right panel, the line represents the median of that group and the percentage represents the portion of cells showing an MFI value > 0. Dye-only ctrl, no EVs was added in the EV dye labeling step. ***, *P* < 0.001; ns, not significant. **D,** Time-dependent uptake of MDA-MB-231 EVs by human lung fibroblasts. An equal amount of EVs (10 µg/mL) were added to the cells for the indicated periods of incubation. Results are presented as in C. Left: *n* = 3 independent wells; right: *n* > 7,000 cells. “1 (on ice)”, cells were incubated with EVs on ice for 1 hour. ***, *P* < 0.001. **E,** Representative images from dose-dependent and time-dependent experiments. Images were captured at 10 ×. Green, CFSE; yellow, DiI; blue, Hoechst 33342. Scale bar, 100 µm.

To establish a method for quantitative measurements of EV uptake by individual cells, we used a lipophilic fluorescent dye (DiI) to label MDA-MB-231 EVs that were subsequently incubated with normal human lung fibroblasts (Fig. 1A). The fibroblasts were stained with CFSE, which generally labeled the cell body, and also with Hoechst 33342 to visualize the nucleus. The two fluorescent channels together enabled single-cell delineation through drawing the boundary of cells and separating cell clusters (Fig. 1B). By using Nikon's automatic image acquisition system and image processing pipeline, this labeling strategy allows us to quantify EV fluorescence in individual cells to reflect any cellular heterogeneity, especially appropriate for high-content microscopy-assisted assessments of EV uptake. To validate the performance of this high-content microscopy platform in assessing EV uptake, we incubated lung fibroblasts with increasing doses of EVs. The results are presented as the calculated mean value of EV fluorescence intensity per cell in a given well or as individual cells from all measured wells, and in either way showed a dose-dependent increase in the cellular signals of DiI reflective of a dose-dependent increase in EV uptake (Fig. 1C). EV uptake also increased with time in a time course study consisting of 0, 1, 3, and 6 hours of incubation (Fig. 1D). To confirm that the detected EV signals were from the cellular compartment instead of EVs bound to the cellular surface, we measured EV uptake on ice to inhibit the cellular uptake process and did not detect significant EV signals (Fig. 1D). Furthermore, to control for any free DiI micelles, we included a dye-only control (no EV) for every batch of staining and did not detect significant signals (Fig. 1C–E).

### Lung Fibroblasts Take up MDA-MB-231 EVs Through Dynamin- and Caveolae-dependent Endocytosis and Macropinocytosis

**FIGURE 2:**
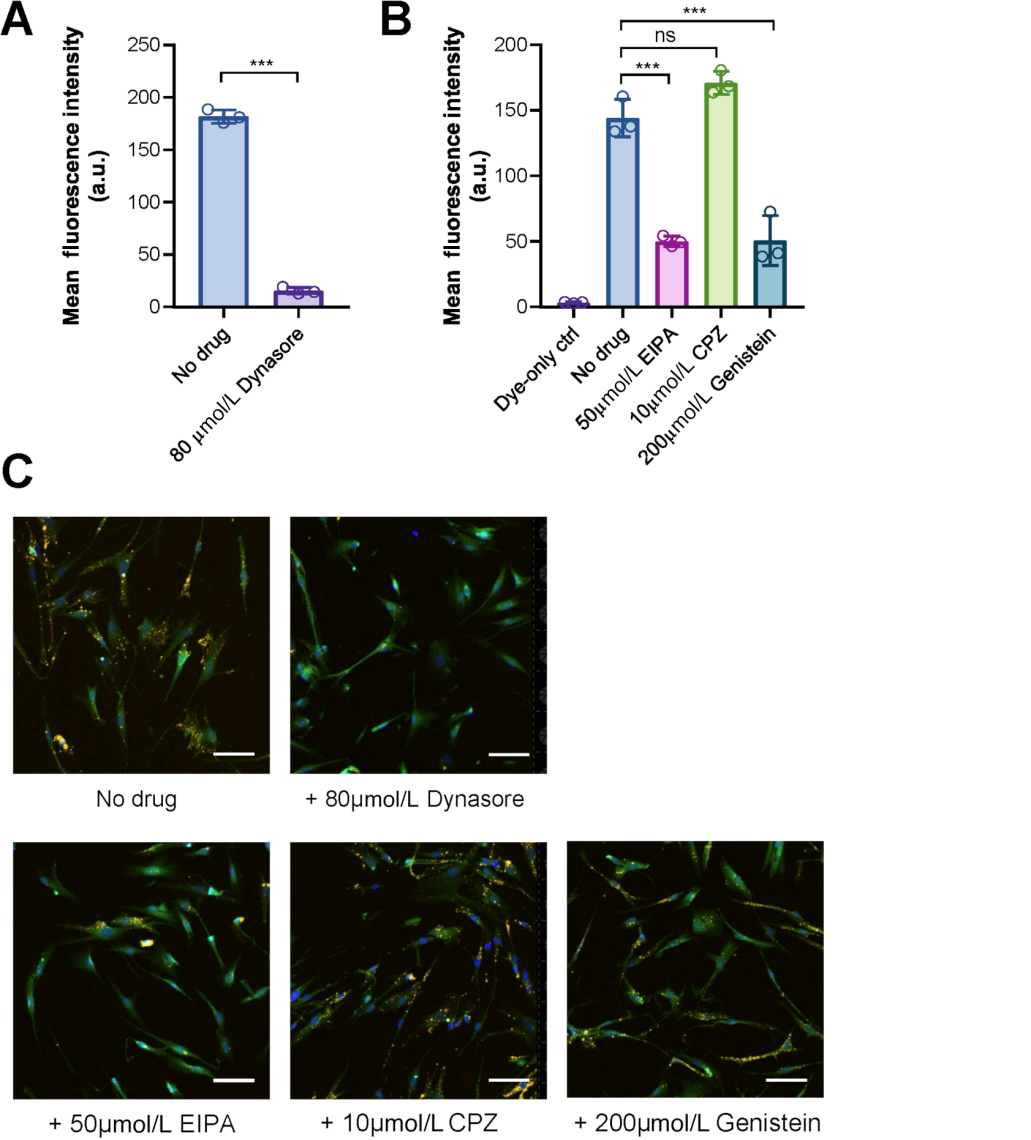
Human lung fibroblasts take up MDA-MB-231 EVs via dynamin- and caveolae-dependent endocytosis and macropinocytosis. **A,** Fibroblasts were pretreated with 80 µmol/L Dynasore or an equal volume of DMSO (as a control) for 24 hours and then incubated with 10 µg/mL EVs for 6 hours in the continuous presence of Dynasore or control (*n* = 3 wells per group; 5 images per well). Data are presented as mean ± SD. ***, *P* < 0.001. **B,** Cells were pretreated with 50 µmol/L EIPA, 10 µmol/L CPZ, or 200 µmol/L Genistein for 24 hours and then incubated with 10 µg/mL EVs for 6 hours in the presence of drug (*n* = 3 wells per group; 5 images per well). Data are presented as mean ± SD. ***, *P* < 0.001; ns, not significant. **C,** Representative images of Dynasore-, EIPA-, CPZ-, or Genistein-treated cells and untreated cells. Images were captured at 10 ×. Green, CFSE; yellow, DiI; blue, Hoechst 33342. Scale bar, 100 µm.

A variety of cell internalization mechanisms have been implicated in EV uptake (17–19). To evaluate which mechanisms are required for the uptake of MDA-MB-231 EVs by lung fibroblasts, we tested the effect of a few established pathway inhibitors. Dynasore, an inhibitor of endocytosis that targets dynamin and suppresses EV uptake in some cells (8, 9), potently inhibited EV uptake in lung fibroblasts by approximately 90% (Fig. 2A). EIPA, an inhibitor of Na^+^/H^+^ exchange and macropinocytosis, as well as Genistein, a tyrosine kinase inhibitor that inhibits caveolae-dependent endocytosis (17, 20, 21), both inhibited EV uptake by approximately 60% (Fig. 2B and C). In contrast, CPZ, a previously reported inhibitor of clathrin-mediated endocytosis (18, 20), had no effect on EV uptake by lung fibroblasts (Fig. 2B and C). Thus, uptake of MDA-MB-231 breast cancer cell-derived EVs by human lung fibroblasts is dynamin dependent, and is mediated by macropinocytosis and caveolae-mediated endocytosis, but not clathrin-mediated endocytosis.

### High-Content Oncology Drug Screen Reveals Compounds that Suppress Cancer EV Uptake in Lung Fibroblasts

We then employed the high-content EV uptake microscopy platform to screen a selected compound library containing 90 FDA-approved anticancer drugs. The compounds were selected from the NCI Development Therapeutics Program's Approved Oncology Drugs Set consisting of 179 anticancer drugs to focus on targeted therapies especially kinase inhibitors. Some drugs that are expected to indirectly affect EV uptake or EV signal intensity in the cells by altering cell viability or proliferation, such as those causing DNA damage or targeting the cell cycle, were not our primary focus and were excluded in this study. In addition to EV uptake screen, we set up parallel plates of cells with compound treatment for assessment of cell viability by MTS assay. Each compound was tested in duplicate wells of cells, using DMSO as a vehicle control and Dynasore as a control compound that is known to potently inhibit EV uptake. For both EV uptake and viability tests, the averaged value from DMSO-treated wells was set as the 100% reference.

**FIGURE 3:**
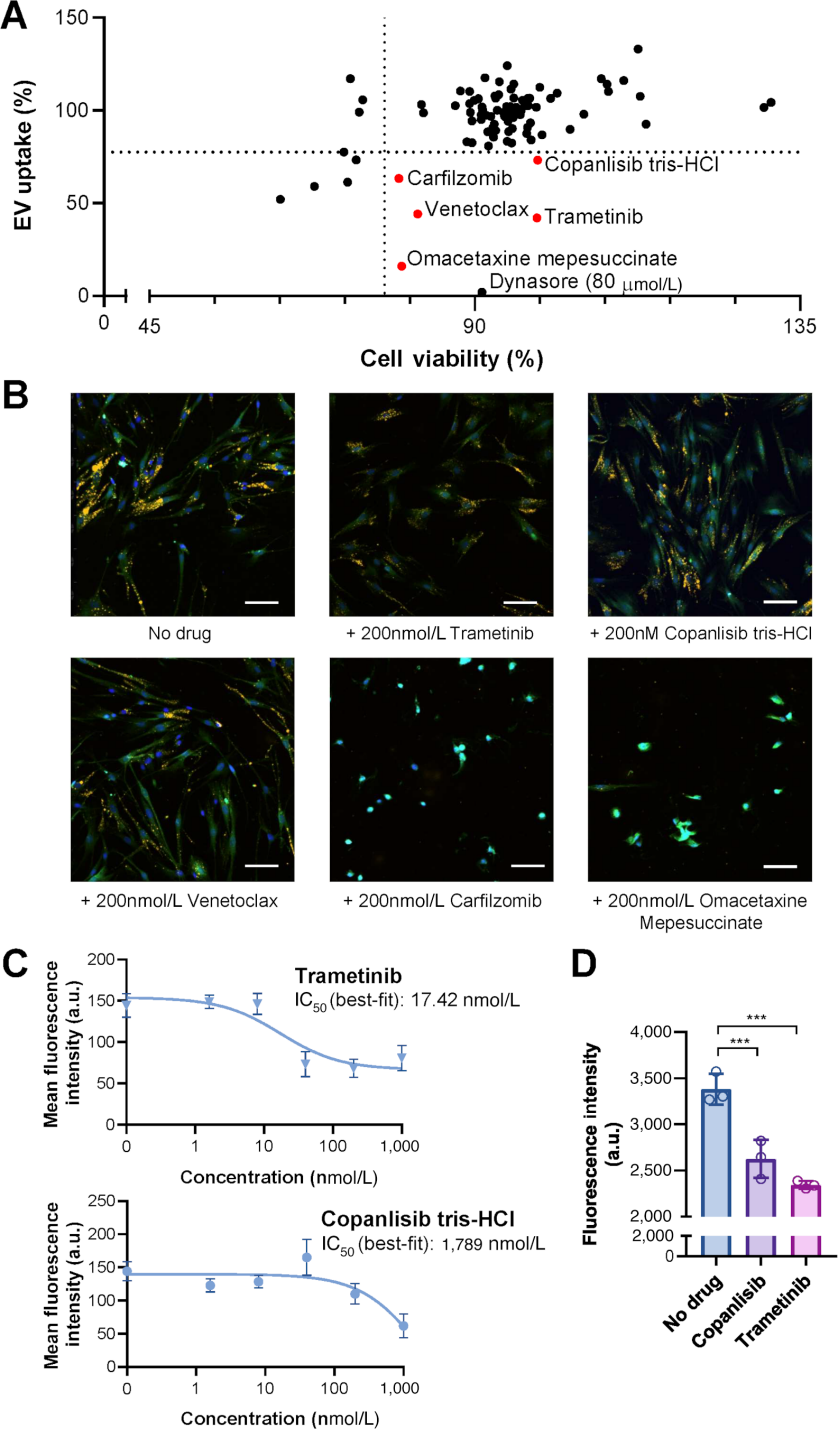
Screening of a targeted library of oncology drugs by high-content microscopy identifies compounds suppressing EV uptake. **A,** Cells were pretreated with drugs (200 nmol/L final concentration) for 24 hours and then either assayed for viability or incubated with 10 µg/mL EV for 6 hours and assayed for EV uptake. Dynasore (80 µmol/L) was also included as a control treatment. For both MTS assay and EV uptake assay, *n* = 2 wells per drug were tested. For EV uptake assay, 5 images per well were captured and analyzed. Each dot represents the mean value of two replicates. Drugs resulting in >75% cell viability and <75% EV uptake efficiency were indicated in red. **B,** Representative images of untreated cells and cells treated with selected drugs. Images were captured at 10 ×. Green, CFSE; yellow, DiI; blue, Hoechst 33342. Scale bar, 100 µm. **C,** Dose-dependent analysis of the effect of Trametinib and Copanlisib. Experiments were carried out as in A using indicated concentrations of the drug. Data are represented as mean ± SD (*n* = 3 wells per group; 5 images per well). The best-fit IC_50_ of each drug is indicated. **D,** Validation of the effect of Trametinib and Copanlisib by flow cytometry. Experiments were carried out as in A but scaled up to a 6-well plate format. Data are represented as mean ± SD (*n* = 3 wells). ***, *P* < 0.001.

On the basis of the screen results, we identified five compounds that caused >25% inhibition of EV uptake while maintaining >75% cell viability at 200 nmol/L (Fig. 3A). These include Trametinib (a MEK1/2 inhibitor), Copanlisib tris-HCl (a PI3K inhibitor), Venetoclax (a Bcl-2 inhibitor), Carfilzomib (a proteosome inhibitor), and Omacetaxine mepesuccinate (a cytotoxic alkaloid; Fig. 3A). Although Carfilzomib and Omacetaxine mepesuccinate passed the cell viability cutoff (>75%) in MTS assay, cells treated with these two drugs showed abnormal morphology including low confluency, loose attachment to the plate, rounded shape, and slow division rate, indicating toxicity (Fig. 3B). We therefore removed Carfilzomib and Omacetaxine mepesuccinate from our list of compounds for further investigation. The inconsistency between results from MTS assay and morphologic assessment could be related to residual metabolic activity of cells, delayed cell death kinetics, and the limited sensitivity of the MTS assay. In addition, we decided not to follow Venetoclax because its primary effect is to induce apoptosis, which could confound the EV uptake result. As such, the oncology drug screen revealed two compounds that suppressed MDA-MB-231 EV uptake by lung fibroblasts without affecting cell viability, Trametinib that targets MEK1/2 and Copanlisib tris-HCl that targets PI3K.

We next determined the effect of varying doses of Trametinib and Copanlisib on EV uptake. Both drugs inhibited EV uptake in a dose-dependent manner (Fig. 3C). Compared with Copanlisib, Trametinib is more potent in inhibiting EV uptake, reaching a plateau of approximately 50% inhibition at only 40 nmol/L. In contrast, Copanlisib caused approximately 50% inhibition at 1,000 nmol/L (Fig. 3C). As a second method to validate the effect of Copanlisib and Trametinib on EV uptake, we measured EV uptake by flow cytometry using DiO as the EV-labeling dye. Consistent with the results of microscopy-assisted EV uptake assessment, flow cytometry also showed that treatment with Copanlisib or Trametinib at 200 nmol/L significantly decreased EV uptake in fibroblasts (Fig. 3D).

**FIGURE 4:**
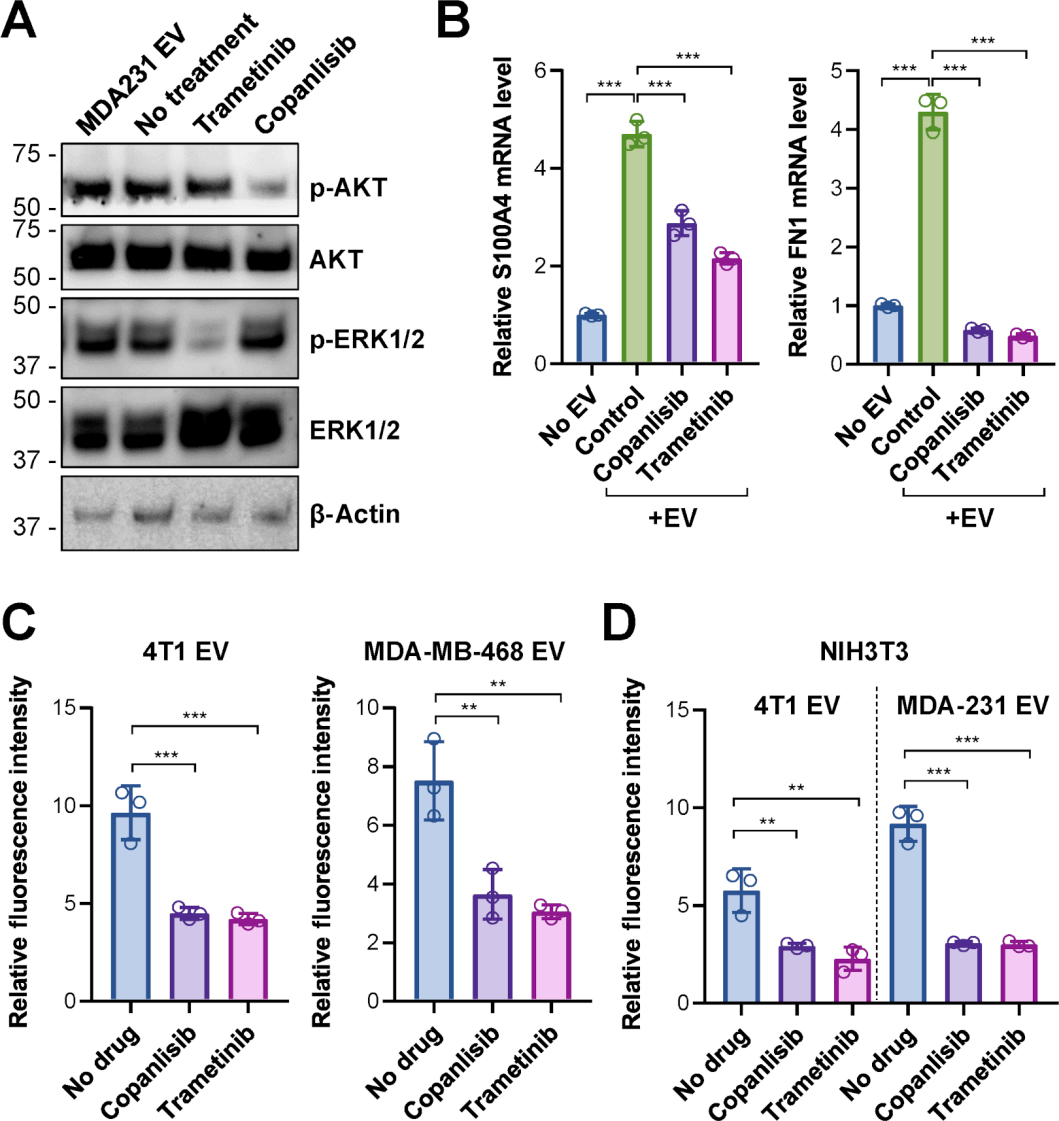
Pathway inhibitors Trametinib and Copanlisib inhibit the uptake and effect of EVs from various breast cancer cells. **A,** Western blots of lung fibroblasts treated with 200 nmol/L of Trametinib or Copanlisib or with 10 µg/mL EV for 24 hours. **B,** qRT-PCR–determined mRNA levels of S100A4 and FN1 with or without MDA-MB-231 EV treatment for 48 hours. Trametinib and Copanlisib were added together with EVs when indicated. Data were normalized to GAPDH. **C,** Lung fibroblasts were pretreated with Trametinib or Copanlisib (200 mmol/L) for 24 hours before Lck-GFP–labeled EVs derived from 4T1 or MDA-MB-468 cells were added and incubated for 6 hours. EV uptake was assessed and compared with DMSO-treated (No drug) control group. **D,** NIH3T3 fibroblasts were pretreated with Trametinib or Copanlisib before Lck-GFP–labeled EVs derived from 4T1 or MDA-MB-231 cells were added. Data are represented as mean ± SD (*n* = 3 wells). **, *P* < 0.01; ***, *P* < 0.001.

Western blots confirmed the inhibition of AKT phosphorylation and ERK1/2 phosphorylation by Copanlisib and Trametinib, respectively, at 200 nmol/L, whereas treatment with MDA-MB-231 EVs did not significantly alter these pathways (Fig. 4A). MDA-MB-231 EVs induced the expression of S100A4 and FN1 in lung fibroblasts (Fig. 4B), which is consistent with other reports identifying these genes as markers for EV-induced premetastatic niches in the lungs (12, 22). The effect of EVs was diminished by treating the fibroblasts with Trametinib or Copanlisib (Fig. 4B). Using EVs from 4T1 and MDA-MB-468 breast cancer cells engineered to overexpress a membrane-targeted Lck-GFP (13), we confirmed that lung fibroblast uptake of EVs from these additional breast cancer cell lines was also inhibited by Trametinib and Copanlisib (Fig. 4C).

### Trametinib and Copanlisib Inhibit Macropinocytosis-mediated EV Uptake

**FIGURE 5:**
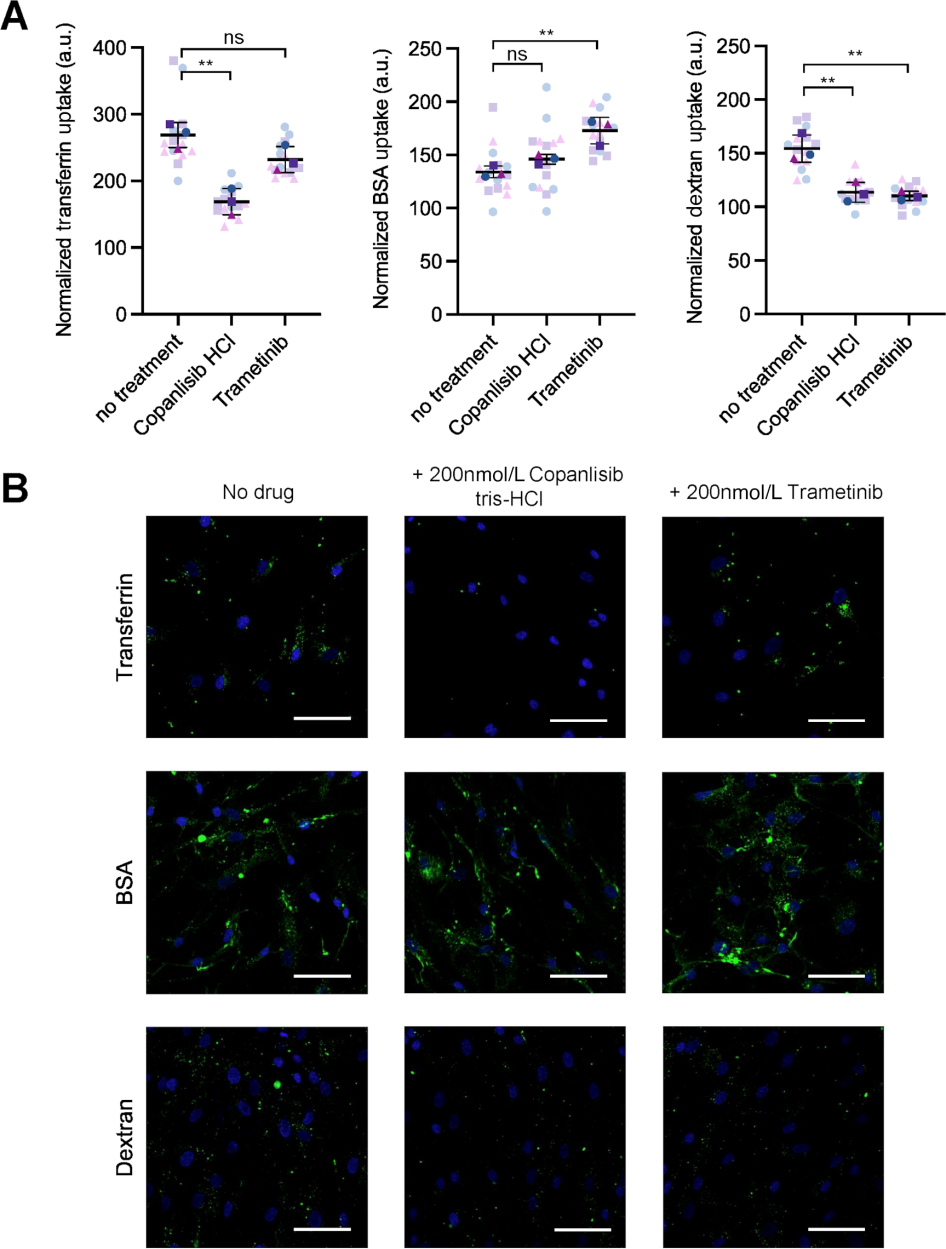
Trametinib interferes with macropinocytosis in lung fibroblasts while Copanlisib interferes with both macropinocytosis and clathrin-mediated endocytosis. **A,** Quantification of the cellular uptake of indicated markers with or without Copanlisib or Trametinib treatment. Data are presented as mean ± SD (*n* = 3 wells per group; 5 images per well). Faint symbols represent all 15 images per group. Dark symbols represent the mean value from each well. **, *P* < 0.01; ns, not significant. **B,** Representative images of the cellular uptake of indicated markers. Images were captured at 10 ×. Green, FITC-transferrin, Alexa488-BSA or FITC-dextran; blue, Hoechst 33342. Scale bar, 100 µm.

To understand which cell uptake pathways Trametinib and Copanlisib interfere with, we assessed the uptake of three pathway markers under the treatment of these drugs. Transferrin, an iron-binding protein, is taken up into cells through clathrin-coated pits and vesicles via receptor-mediated endocytosis and is frequently as a marker in studies of clathrin-mediated endocytosis (23). Dextran, a well-characterized marker for macropinocytosis (24), and BSA, a commonly used marker for caveolae-mediated endocytosis (25, 26), were also included. Consistent with the specificity of the involved cellular uptake pathways, uptake of dextran was significantly inhibited by EIPA, the macropinocytosis inhibitor, but not by CPZ or Genistein. Uptake of transferrin was most significantly inhibited by CPZ, the inhibitor of clathrin-mediated endocytosis. Uptake of BSA was partially inhibited by all three inhibitors, with Genistein showing the strongest inhibitory effect (Supplementary Fig. S2). Using these markers indicating different cell uptake pathways, we observed that Copanlisib significantly inhibited the uptake of transferrin and dextran, whereas Trametinib significantly inhibited the uptake of dextran but increased BSA uptake (Fig. 5A). Thus, we concluded that Trametinib mainly inhibited macropinocytosis while Copanlisib inhibited both macropinocytosis and clathrin-mediated endocytosis. As we show earlier that MDA-MB-231 EVs enter lung fibroblasts mainly through macropinocytosis and caveolae-mediated endocytosis, we further concluded that Trametinib and Copanlisib mainly block macropinocytosis-mediated EV entry. It has been previously reported that PI3K inhibitors suppress macropinocytosis and phagocytosis, thereby blocking EV uptake in a variety of cells (18, 27), whereas the role of MEK1/2 has not been elucidated.

### MEK2, but not MEK1, Mediates EV Uptake in Lung Fibroblasts

**FIGURE 6:**
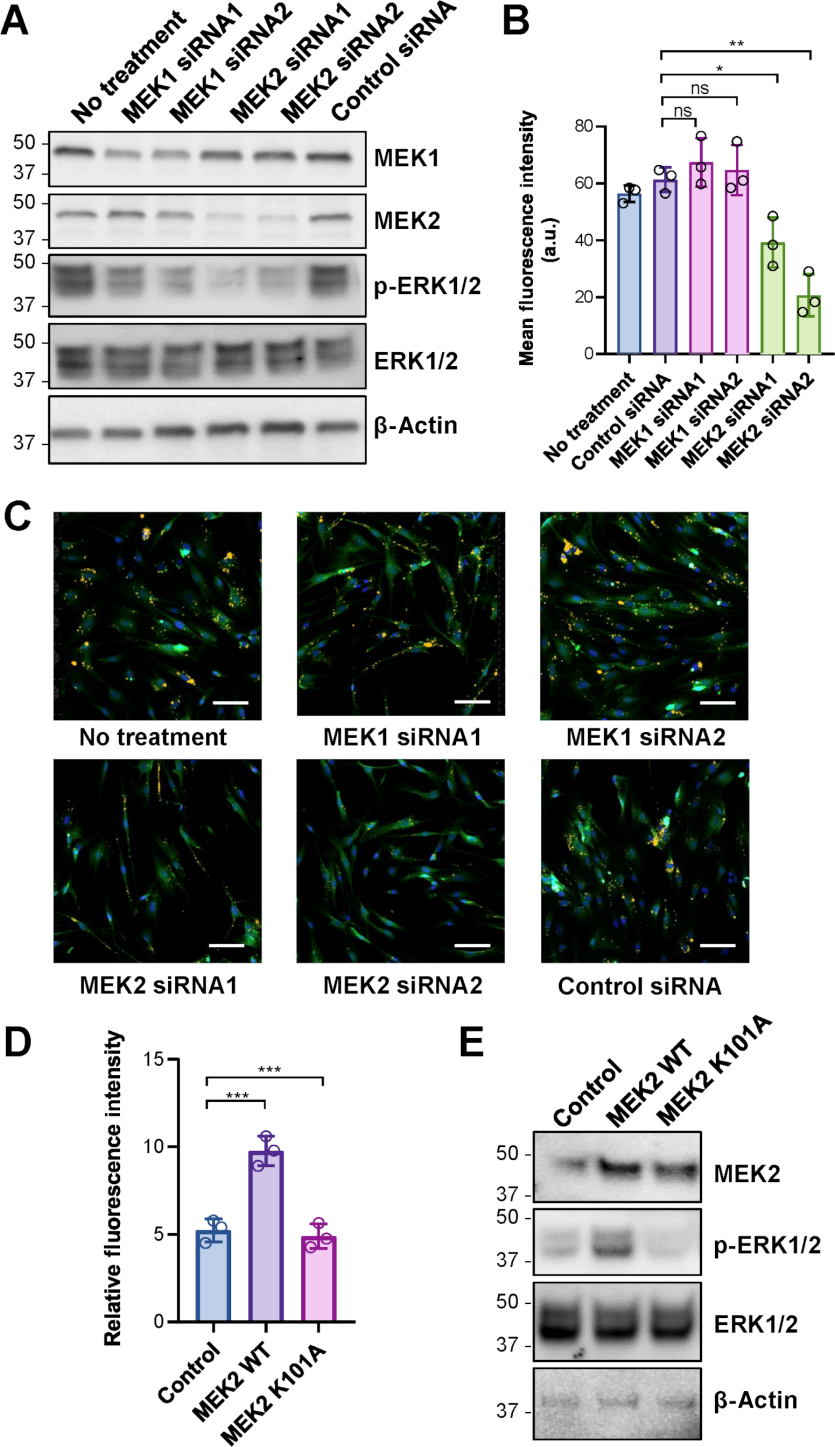
MDA-MB-231 EV uptake by lung fibroblasts requires MEK2 but not MEK1. **A,** Fibroblasts were transfected with siRNAs against MEK1 or MEK2 (two independent siRNAs used for each gene), or with a control siRNA, at 48 hours before EVs were added for an incubation of 6 hours (*n* = 3 wells per group). **B,** Western blots of cells transfected with indicated siRNAs showing the gene knockdown efficiency of MEK1/2. **C,** Representative images of untreated cells and cells transfected with indicated siRNAs. Images were captured at 10 ×. Green, CFSE; yellow, DiI; blue, Hoechst 33342. Scale bar, 100 µm. **D,** Fibroblasts were transfected with an overexpression plasmid encoding wild-type (WT) or catalytically inactive (K101A) MEK2, or with the empty vector. Forty-eight hours later, cells were incubated with MDA-MB-231 EVs for 6 hours (*n* = 3 wells per group). **E,** Western blots of cells transfected with indicated overexpression plasmids. Data are presented as mean ± SD. *, *P* < 0.05; **, *P* < 0.01; ***, *P* < 0.001; ns, not significant.

It has caught our attention that Trametinib was not the only MEK1/2 inhibitor in our selected compound library. The other three MEK1/2 inhibitors in the library, namely Cobimetinib, Selumetinib, and Binimetinib, did not suppress EV uptake by >25%. Interestingly, Cobimetinib exhibits a specificity against MEK1 (IC_50_ against MEK1 = 0.95 nmol/L in comparison with IC_50_ against MEK2 = 199 nmol/L; refs. 28, 29), whereas Trametinib potently inhibits both MEK1 and MEK2 with IC_50_ < 1 nmol/L (30). Selumetinib and Binimetinib are designed to target both MEK1 and MEK2 but their IC_50_ against MEK1/2 are >10-fold higher comparing with Trametinib (31–33). Thus, we hypothesized that MEK2 plays a more important role than MEK1 in EV uptake by lung fibroblasts. To test this hypothesis, we used siRNA to individually knock down MEK1 and MEK2 in lung fibroblasts and assessed EV uptake by comparing with the control siRNA treatment group. Western blot analysis confirmed significant decreases of MEK1 or MEK2 proteins upon knockdown of the corresponding genes and the consequent reductions of ERK1/2 phosphorylation (Fig. 6A). Importantly, although lung fibroblasts express both MEK1 and MEK2 proteins at significant levels, only knockdown of MEK2 but not MEK1 led to significant decreases in EV uptake (Fig. 6B and C). Overexpression of wild-type MEK2, but not a catalytically inactive mutant, increased EV uptake (Fig. 6D and E). The results collectively suggest that the MEK2-specific signaling pathway plays a critical role in MDA-MB-231 EV uptake by lung fibroblasts.

## Discussion

Cellular uptake of EVs is a highly dynamic event that can be influenced by multiple cell-intrinsic and -extrinsic factors. Although the detrimental effect of cancer cell-derived EVs has been widely demonstrated, development of therapeutic strategies to block the entry of pathogenic EVs into healthy cells has been hindered by the poor understanding of mechanisms controlling EV uptake. Here, by screening a targeted library of approved oncology drugs, we aim to identify those anticancer drugs that also inhibit the uptake of cancer cell-derived EVs for rapid translation, by guiding selection of a more effective anticancer drug regimen and expanding the indications for some drugs. We employed a high-content microscopy platform to quantitatively measure EV uptake at the individual cell level with a relatively high throughput. Compared with flow cytometry, which can also detect EV fluorescence in individual cells, the high-content microscopy platform not only provides a much higher throughput but also avoids the need to detach cells from the culture plate, thereby maximally preserving the integrity of cells and preventing potential leakage of cellular EV signals. Our results indicate that MEK2, but not MEK1, mediates the uptake of breast cancer cell-derived EVs by normal lung fibroblasts through micropinocytosis, and that inhibition of MEK2 could be a potential strategy to protect lung fibroblasts from cancer-secreted EVs. In the compound screen, we tested all compounds at 200 nmol/L. It is possible that inhibitors targeting the same kinase exhibit different potency at this concentration and therefore show different effect on EV uptake. Nevertheless, the role of MEK2 in EV uptake was confirmed by gene knockdown and overexpression in our study.

Recent studies have elucidated multiple pathways in EVs biogenesis and secretion (34). The mechanisms of EV uptake may be specific to the type of recipient cells and to the source of EVs. HeLa cell uptake of EVs from A431 epidermoid carcinoma cells depends on clathrin-independent endocytosis and macropinocytosis (17). In contrast, PC12 rat pheochromocytoma cells take up EVs derived from the same cells via clathrin-mediated endocytosis and macropinocytosis (18). Ligand-induced activation of EGFR promotes, while EGFR signaling blockage inhibits, the autocrine uptake of oral squamous cell carcinoma cell-derived EVs through regulating macropinocytosis (19). Here we find that lung fibroblasts take up MDA-MB-231 breast cancer cell-derived EVs mainly through macropinocytosis and clathrin-independent, caveolae-mediated endocytosis, and that MEK inhibition by Trametinib mainly suppresses macropinocytosis. We noted that Trametinib did not fully inhibit EV uptake but only resulted in a maximal inhibition of approximately 50% (Fig. 4C). It is possible that the remaining EV uptake events that were resistant to MEK inhibition were mediated by other uptake mechanisms such as caveolae-mediated endocytosis.

The MEK1/2 kinases have been attractive targets for cancer therapy especially for cancers associated with aberrant activation of the RAS-RAF-MEK-ERK pathway, including melanomas and non–small cell lung cancer (NSCLC; ref. 35). The MEK1/2 kinases are a key component of the signaling cascades of many surface receptors and mediates the malignant cell transformation by mutated EGFR and KRAS. Because the MEK kinases are the only known activator of ERK1/2, and because ERK1/2 are the only known substrates of MEK and play a critical role in cell proliferation and tumorigenesis (35, 36), inhibition of MEK has been considered a relatively specific and effective approach in cancer therapy. MEK1 and MEK2 share approximately 80% amino acid identity but have unique sequences in their C-terminal domains. MEK2 has been shown to be more potent in phosphorylating recombinant ERK (37, 38). MEK1 and MEK2 seem to be differentially regulated by upstream stimulators at least in some cellular settings (39). The crystal structures of MEK1 and MEK2 reveal that the two kinases each have a unique inhibitor-binding pocket adjacent to the MgATP-binding site, which allows selective inhibition by certain inhibitors (40).

Trametinib is an oral allosteric inhibitor of MEK1/2 that potently inhibits both kinases with an IC_50_ of 0.7–0.9 nmol/L (30). Trametinib, as a monotherapy, first received its FDA approval for the treatment of some melanomas carrying certain BRAF mutations and was subsequently approved as a combinational therapy for some solid tumors including gliomas and NSCLC. It also shows promise as a targeted therapy for KRAS-mutated NSCLC (41) and for rare breast cancers carrying BRAF mutations (42). Our findings herein further suggest inhibitors of MEK kinases, especially MEK2, might confer a protective effect on lung fibroblasts by preventing the entry of some cancer cell-derived EVs. This may support the utility of MEK inhibitors as a combinational therapy for the prevention or treatment of breast cancer metastasis to the lungs. Further studies are warranted to determine the *in vivo* effect of MEK2 inhibitors on EV uptake and cancer metastasis using preclinical models and lung specimens from patients who have been treated with MEK inhibitors.

## Supplementary Material

Figure S1 Supplementary Figure S1 shows characterization of EVs. (A) Western blots of whole cell lysates (WCL) and EVs from MDA-MB-231 showing EV markers and a Golgi marker (GM130, as a negative control for EV-specific proteins). (B) Nanoparticle tracking analysis (NTA) of MDA-MB-231 EVs showing size distribution (n=3 biological replicates). Data are presented as mean ± standard error of the mean (SEM).

Figure S2 Supplementary Figure S2 shows the effect of the inhibitors of different cell uptake pathways on marker uptake. Lung fibroblasts were pre-treated with 50 μM EIPA, 10 μM CPZ, or 200 μM Genistein for 24 h and then incubated with FITC-transferrin, Alexa488-BSA or FITC-dextran for 6 h in the continuous presence of the inhibitor (n=3 wells per group; 5 images per well). Data are presented as mean ± SD. *, p<0.05; **, p<0.01; ***, p<0.001; ns, not significant.

Table S1 Supplementary Table S1 shows the compounds tested in the screen.

Table S2 Supplementary Table S2 shows the antibodies used in this study.

## References

[1] Becker A , Thakur BK, Weiss JM, Kim HS, Peinado H, Lyden D. Extracellular vesicles in cancer: cell-to-cell mediators of metastasis. Cancer Cell 2016;30:836–48. 27960084 10.1016/j.ccell.2016.10.009 PMC5157696

[2] Wang SE . Extracellular vesicles and metastasis. Cold Spring Harb Perspect Med 2020;10:a037275. 31570387 10.1101/cshperspect.a037275 PMC7328450

[3] Mitchell PS , Parkin RK, Kroh EM, Fritz BR, Wyman SK, Pogosova-Agadjanyan EL, . Circulating microRNAs as stable blood-based markers for cancer detection. Proc Natl Acad Sci U S A 2008;105:10513–8. 18663219 10.1073/pnas.0804549105 PMC2492472

[4] Wu X , Somlo G, Yu Y, Palomares MR, Li AX, Zhou W, . *De novo* sequencing of circulating miRNAs identifies novel markers predicting clinical outcome of locally advanced breast cancer. J Transl Med 2012;10:42. 22400902 10.1186/1479-5876-10-42 PMC3342150

[5] Wang SE . Extracellular vesicles in cancer therapy. Semin Cancer Biol 2022;86:296–309. 35688334 10.1016/j.semcancer.2022.06.001 PMC10431950

[6] Zhou W , Fong MY, Min Y, Somlo G, Liu L, Palomares MR, . Cancer-secreted miR-105 destroys vascular endothelial barriers to promote metastasis. Cancer Cell 2014;25:501–15. 24735924 10.1016/j.ccr.2014.03.007 PMC4016197

[7] Fong MY , Zhou W, Liu L, Alontaga AY, Chandra M, Ashby J, . Breast-cancer-secreted miR-122 reprograms glucose metabolism in premetastatic niche to promote metastasis. Nat Cell Biol 2015;17:183–94. 25621950 10.1038/ncb3094 PMC4380143

[8] Kawamoto T , Ohga N, Akiyama K, Hirata N, Kitahara S, Maishi N, . Tumor-derived microvesicles induce proangiogenic phenotype in endothelial cells via endocytosis. PLoS One 2012;7:e34045. 22479517 10.1371/journal.pone.0034045 PMC3316594

[9] Yan W , Wu X, Zhou W, Fong MY, Cao M, Liu J, . Cancer-cell-secreted exosomal miR-105 promotes tumour growth through the MYC-dependent metabolic reprogramming of stromal cells. Nat Cell Biol 2018;20:597–609. 29662176 10.1038/s41556-018-0083-6 PMC5920728

[10] Casamento A , Boucrot E. Molecular mechanism of fast endophilin-mediated endocytosis. Biochem J 2020;477:2327–45. 32589750 10.1042/BCJ20190342 PMC7319585

[11] Preta G , Cronin JG, Sheldon IM. Dynasore – not just a dynamin inhibitor. Cell Commun Signal 2015;13:24. 25889964 10.1186/s12964-015-0102-1 PMC4396812

[12] Hoshino A , Costa-Silva B, Shen TL, Rodrigues G, Hashimoto A, Tesic Mark M, . Tumour exosome integrins determine organotropic metastasis. Nature 2015;527:329–35. 26524530 10.1038/nature15756 PMC4788391

[13] Yan W , Cao M, Ruan X, Jiang L, Lee S, Lemanek A, . Cancer-cell-secreted miR-122 suppresses O-GlcNAcylation to promote skeletal muscle proteolysis. Nat Cell Biol 2022;24:793–804. 35469018 10.1038/s41556-022-00893-0 PMC9107513

[14] Uphoff CC , Drexler HG. Detecting mycoplasma contamination in cell cultures by polymerase chain reaction. Methods Mol Biol 2011;731:93–103. 21516400 10.1007/978-1-61779-080-5_8

[15] Fong MY , Yan W, Ghassemian M, Wu X, Zhou X, Cao M, . Cancer-secreted miRNAs regulate amino-acid-induced mTORC1 signaling and fibroblast protein synthesis. EMBO Rep 2021;22:e51239. 33345445 10.15252/embr.202051239 PMC7857427

[16] Thery C , Witwer KW, Aikawa E, Alcaraz MJ, Anderson JD, Andriantsitohaina R, . Minimal information for studies of extracellular vesicles 2018 (MISEV2018): a position statement of the International Society for Extracellular Vesicles and update of the MISEV2014 guidelines. J Extracell Vesicles 2018;7:1535750. 30637094 10.1080/20013078.2018.1535750 PMC6322352

[17] Costa Verdera H , Gitz-Francois JJ, Schiffelers RM, Vader P. Cellular uptake of extracellular vesicles is mediated by clathrin-independent endocytosis and macropinocytosis. J Control Release 2017;266:100–8. 28919558 10.1016/j.jconrel.2017.09.019

[18] Tian T , Zhu YL, Zhou YY, Liang GF, Wang YY, Hu FH, . Exosome uptake through clathrin-mediated endocytosis and macropinocytosis and mediating miR-21 delivery. J Biol Chem 2014;289:22258–67. 24951588 10.1074/jbc.M114.588046 PMC4139237

[19] Sasabe E , Tomomura A, Liu H, Sento S, Kitamura N, Yamamoto T. Epidermal growth factor/epidermal growth factor receptor signaling blockage inhibits tumor cell-derived exosome uptake by oral squamous cell carcinoma through macropinocytosis. Cancer Sci 2022;113:609–21. 34874595 10.1111/cas.15225 PMC8819298

[20] Khalil IA , Kogure K, Akita H, Harashima H. Uptake pathways and subsequent intracellular trafficking in nonviral gene delivery. Pharmacol Rev 2006;58:32–45. 16507881 10.1124/pr.58.1.8

[21] Pelkmans L , Puntener D, Helenius A. Local actin polymerization and dynamin recruitment in SV40-induced internalization of caveolae. Science 2002;296:535–9. 11964480 10.1126/science.1069784

[22] Medeiros B , Goodale D, Postenka C, Lowes LE, Kiser P, Hearn S, . Triple-negative primary breast tumors induce supportive premetastatic changes in the extracellular matrix and soluble components of the lung microenvironment. Cancers 2020;12:172. 31936750 10.3390/cancers12010172 PMC7016570

[23] Sorkin A , von Zastrow M. Endocytosis and signalling: intertwining molecular networks. Nat Rev Mol Cell Biol 2009;10:609–22. 19696798 10.1038/nrm2748 PMC2895425

[24] Swanson JA , Watts C. Macropinocytosis. Trends Cell Biol 1995;5:424–8. 14732047 10.1016/s0962-8924(00)89101-1

[25] Singh RD , Puri V, Valiyaveettil JT, Marks DL, Bittman R, Pagano RE. Selective caveolin-1-dependent endocytosis of glycosphingolipids. Mol Biol Cell 2003;14:3254–65. 12925761 10.1091/mbc.E02-12-0809 PMC181565

[26] Schubert W , Frank PG, Razani B, Park DS, Chow CW, Lisanti MP. Caveolae-deficient endothelial cells show defects in the uptake and transport of albumin *in vivo*. J Biol Chem 2001;276:48619–22. 11689550 10.1074/jbc.C100613200

[27] Mulcahy LA , Pink RC, Carter DRF. Routes and mechanisms of extracellular vesicle uptake. J Extracell Vesicles 2014;3. 10.3402/jev.v3.24641 PMC4122821 25143819

[28] Cheng Y , Tian H. Current development status of MEK inhibitors. Molecules 2017;22:1551. 28954413 10.3390/molecules22101551 PMC6151813

[29] Rice KD , Aay N, Anand NK, Blazey CM, Bowles OJ, Bussenius J, . Novel carboxamide-based allosteric MEK inhibitors: discovery and optimization efforts toward XL518 (GDC-0973). ACS Med Chem Lett 2012;3:416–21. 24900486 10.1021/ml300049d PMC4025802

[30] Gilmartin AG , Bleam MR, Groy A, Moss KG, Minthorn EA, Kulkarni SG, . GSK1120212 (JTP-74057) is an inhibitor of MEK activity and activation with favorable pharmacokinetic properties for sustained *in vivo* pathway inhibition. Clin Cancer Res 2011;17:989–1000. 21245089 10.1158/1078-0432.CCR-10-2200

[31] Han J , Liu Y, Yang S, Wu X, Li H, Wang Q. MEK inhibitors for the treatment of non-small cell lung cancer. J Hematol Oncol 2021;14:1. 33402199 10.1186/s13045-020-01025-7 PMC7786519

[32] Woodfield SE , Zhang L, Scorsone KA, Liu Y, Zage PE. Binimetinib inhibits MEK and is effective against neuroblastoma tumor cells with low NF1 expression. BMC Cancer 2016;16:172. 26925841 10.1186/s12885-016-2199-z PMC4772351

[33] Yeh TC , Marsh V, Bernat BA, Ballard J, Colwell H, Evans RJ, . Biological characterization of ARRY-142886 (AZD6244), a potent, highly selective mitogen-activated protein kinase kinase 1/2 inhibitor. Clin Cancer Res 2007;13:1576–83. 17332304 10.1158/1078-0432.CCR-06-1150

[34] Colombo M , Moita C, van Niel G, Kowal J, Vigneron J, Benaroch P, . Analysis of ESCRT functions in exosome biogenesis, composition and secretion highlights the heterogeneity of extracellular vesicles. J Cell Sci 2013;126:5553–65. 24105262 10.1242/jcs.128868

[35] Heigener DF , Gandara DR, Reck M. Targeting of MEK in lung cancer therapeutics. Lancet Respir Med 2015;3:319–27. 25801412 10.1016/S2213-2600(15)00026-0

[36] Roskoski R Jr . ERK1/2 MAP kinases: structure, function, and regulation. Pharmacol Res 2012;66:105–43. 22569528 10.1016/j.phrs.2012.04.005

[37] Zheng CF , Guan KL. Properties of MEKs, the kinases that phosphorylate and activate the extracellular signal-regulated kinases. J Biol Chem 1993;268:23933–9. 8226933

[38] Zheng CF , Guan KL. Cloning and characterization of two distinct human extracellular signal-regulated kinase activator kinases, MEK1 and MEK2. J Biol Chem 1993;268:11435–9. 8388392

[39] Xu S , Khoo S, Dang A, Witt S, Do V, Zhen E, . Differential regulation of mitogen-activated protein/ERK kinase (MEK)1 and MEK2 and activation by a Ras-independent mechanism. Mol Endocrinol 1997;11:1618–25. 9328344 10.1210/mend.11.11.0010

[40] Ohren JF , Chen H, Pavlovsky A, Whitehead C, Zhang E, Kuffa P, . Structures of human MAP kinase kinase 1 (MEK1) and MEK2 describe novel noncompetitive kinase inhibition. Nat Struct Mol Biol 2004;11:1192–7. 15543157 10.1038/nsmb859

[41] Blumenschein GR Jr , Smit EF, Planchard D, Kim DW, Cadranel J, De Pas T, . A randomized phase II study of the MEK1/MEK2 inhibitor trametinib (GSK1120212) compared with docetaxel in KRAS-mutant advanced non-small-cell lung cancer (NSCLC)^†^. Ann Oncol 2015;26:894–901. 25722381 10.1093/annonc/mdv072 PMC4855243

[42] Seo T , Noguchi E, Yoshida M, Mori T, Tanioka M, Sudo K, . Response to dabrafenib and trametinib of a patient with metaplastic breast carcinoma harboring a BRAF V600E mutation. Case Rep Oncol Med 2020;2020:2518383. 32206360 10.1155/2020/2518383 PMC7079252

