# Supplementary material for: Lung Fibroblasts Take up Breast Cancer Cell-derived Extracellular Vesicles Partially Through MEK2-dependent Macropinocytosis
Source: Cancer Res Commun. 2024 Jan 22;4(1):170–81. doi: 10.1158/2767-9764.CRC-23-0316 (PMC10802141; doi:10.1158/2767-9764.CRC-23-0316)
Supplement: Figure S1 — Supplementary Figure S1 shows characterization of EVs. (A) Western blots of whole cell lysates (WCL) and EVs from MDA-MB-231 showing EV markers and a Golgi marker (GM130, as a negative control for EV-specific proteins). (B) Nanoparticle tracking analysis (NTA) of MDA-MB-231 EVs showing size distribution (n=3 biological replicates). Data are presented as mean ± standard error of the mean (SEM). [file crc-23-0316-s01.pdf]

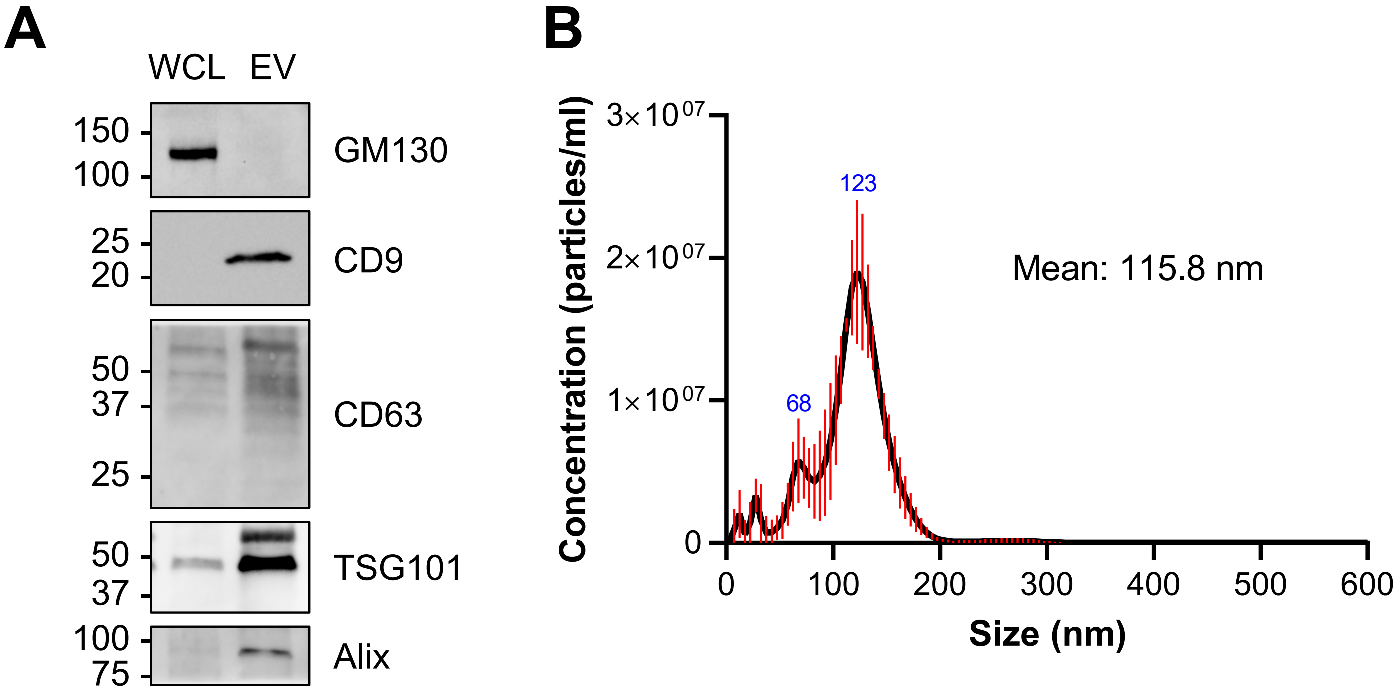

Supplementary Fig. S1. Characterization of EVs. (A) Western blots of whole cell lysates (WCL) and EVs from MDA-MB-231 showing EV markers and a Golgi marker (GM130, as a negative control for EV-specific proteins). (B) Nanoparticle tracking analysis (NTA) of MDA-MB-231 EVs showing size distribution (n=3 biological replicates). Data are presented as mean ± standard error of the mean (SEM).
