# Supplementary material for: Lung Fibroblasts Take up Breast Cancer Cell-derived Extracellular Vesicles Partially Through MEK2-dependent Macropinocytosis
Source: Cancer Res Commun. 2024 Jan 22;4(1):170–81. doi: 10.1158/2767-9764.CRC-23-0316 (PMC10802141; doi:10.1158/2767-9764.CRC-23-0316)
Supplement: Figure S2 — Supplementary Figure S2 shows the effect of the inhibitors of different cell uptake pathways on marker uptake. Lung fibroblasts were pre-treated with 50 μM EIPA, 10 μM CPZ, or 200 μM Genistein for 24 h and then incubated with FITC-transferrin, Alexa488-BSA or FITC-dextran for 6 h in the continuous presence of the inhibitor (n=3 wells per group; 5 images per well). Data are presented as mean ± SD. *, p<0.05; **, p<0.01; ***, p<0.001; ns, not significant. [file crc-23-0316-s02.pdf]

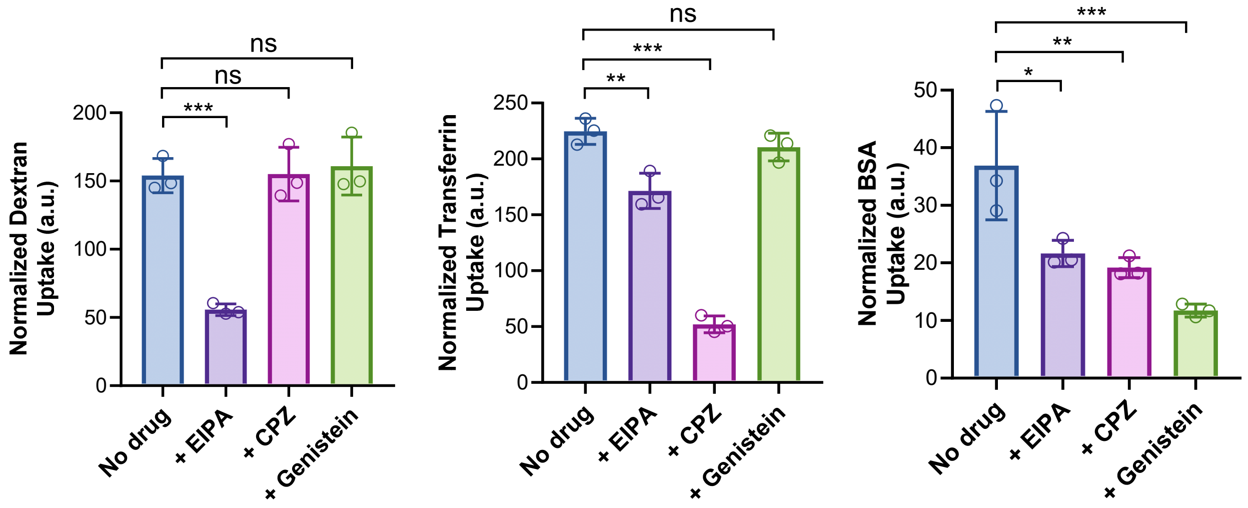

Supplementary Fig. S2. Effect of the inhibitors of different cell uptake pathways on marker uptake. Lung fibroblasts were pre-treated with 50 µM EIPA, 10 µM CPZ, or 200 µM Genistein for 24 h and then incubated with FITC-transferrin, Alexa488-BSA or FITC-dextran for 6 h in the continuous presence of the inhibitor (n=3 wells per group; 5 images per well). Data are presented as mean ± SD. *, $p<0.05$; **, $p<0.01$; ***, $p<0.001$; ns, not significant.
